# Onset of a pandemic: characterizing the initial phase of the swine flu (H1N1) epidemic in Israel

**DOI:** 10.1186/1471-2334-11-92

**Published:** 2011-04-14

**Authors:** Uri Roll, Rami Yaari, Guy Katriel, Oren Barnea, Lewi Stone, Ella Mendelson, Michal Mendelboim, Amit Huppert

**Affiliations:** 1 Biomathematics Unit, Department of Zoology, Faculty of Life Sciences, Tel-Aviv University, 69978 Tel-Aviv, Israel; 2 The Porter School of Environmental Studies, Tel-Aviv University, 69978 Tel-Aviv, Israel; 3 Central Virology Laboratory, Ministry of Health, Chaim Sheba Medical Center, Tel-Hashomer; 4 School of Public Health, the Sackler Faculty of Medicine, Tel-Aviv University; 5 National Influenza Center, Central Virology Laboratory, Ministry of Health, Chaim Sheba Medical Center, Tel-Hashomer; 6 Center for Risk Analysis, the Gertner Institute, Chaim Sheba Medical Center, Tel Hashomer, Israel

**Keywords:** Epidemiology, H1N1, pandemic influenza, swine-flu

## Abstract

**Background:**

The swine influenza H1N1 first identified in Mexico, spread rapidly across the globe and is considered the fastest moving pandemic in history. The early phase of an outbreak, in which data is relatively scarce, presents scientific challenges on key issues such as: scale, severity and immunity which are fundamental for establishing sound and rapid policy schemes. Our analysis of an Israeli dataset aims at understanding the spatio-temporal dynamics of H1N1 in its initial phase.

**Methods:**

We constructed and analyzed a unique dataset from Israel on all confirmed cases (between April 26 to July 7, 2009), representing most swine flu cases in this period. We estimated and characterized fundamental epidemiological features of the pandemic in Israel (e.g. effective reproductive number, age-class distribution, at-risk social groups, infections between sexes, and spatial dynamics). Contact data collected during this stage was used to estimate the generation time distribution of the pandemic.

**Results:**

We found a low effective reproductive number (*R*_*e *_= 1.06), an age-class distribution of infected individuals (skewed towards ages 18-25), at-risk social groups (soldiers and ultra Orthodox Jews), and significant differences in infections between sexes (skewed towards males). In terms of spatial dynamics, the pandemic spread from the central coastal plain of Israel to other regions, with higher infection rates in more densely populated sub-districts with higher income households.

**Conclusions:**

Analysis of high quality data holds much promise in reducing uncertainty regarding fundamental aspects of the initial phase of an outbreak (e.g. the effective reproductive number R_e_, age-class distribution, at-risk social groups). The formulation for determining the effective reproductive number *R*_*e *_used here has many advantages for studying the initial phase of the outbreak since it neither assumes exponential growth of infectives and is independent of the reporting rate. The finding of a low *R*_*e *_(close to unity threshold), combined with identification of social groups with high transmission rates would have enabled the containment of swine flu during the summer in Israel. Our unique use of contact data provided new insights into the differential dynamics of influenza in different ages and sexes, and should be promoted in future epidemiological studies. Thus our work highlights the importance of conducting a comprehensive study of the initial stage of a pandemic in real time.

## Background

Influenza A of the H1N1 subtype, known as swine flu, was identified as a new strain in Mexico and is now considered the fastest moving pandemic in the history of the world. As it circled the globe it received much attention by the scientific community and policy makers [1]. The early phase of an outbreak presents scientists with challenges on key issues such as: the magnitude of the problem at hand when a disease first appears, population susceptibility, attack rates, and severity of the illness and its symptoms, all of which are uncertain when a new disease first appears. Acting early and establishing rapid policy schemes is crucial. However such urgent decisions have to be made under great uncertainty due to the lack, or poor quality, of data [2].

Several basic characteristics of the swine flu epidemic differentiate it from seasonal influenza. The disease spread during late spring and summer in Northern Hemisphere [3] - a phenomenon that has also been observed in previous pandemics [4]. Also, the age distribution of individuals infected with swine flu deviates from that of seasonal influenza - with more morbidity and mortality amongst young adults [5], again a characteristic noted in previous pandemics [6,7].

This work makes use of the fact that the data gathered in Israel from April 26, 2009 (the first confirmed case) until July 7, 2009, is of great detail. During the first two months of the outbreak the Israeli health authorities attempted to identify and test all cases of people suspected with symptomatic swine flu. Their efforts were aided by the high media impact of the disease and the attentiveness of the general public. There is reason to believe that most symptomatic swine flu cases in Israel over this period were diagnosed by the national surveillance campaign, making the dataset unique in its detail and scope.

The national surveillance also provided contact data that presented a snapshot of the population's infection network. During the time span of this study the number of laboratory confirmed cases in Israel (713) was the third highest in Europe exceeded only by the UK and Spain, which have much larger populations [8]. The prevalence and fatality rate of swine flu in Israel remained high compared to other Mediterranean and European countries.

We examined the spatio-temporal distribution of all cases and sub-groups and various characteristics of the disease. This analysis of the unique Israeli dataset made it possible to elucidate numerous interesting aspects of the initial phase of a pandemic.

## Methods

Our analysis is based only on cases that are laboratory confirmed with swine flu. The first person to be diagnosed in Israel as suffering from swine influenza infection arrived to Israel from Mexico and was hospitalized in the Laniado hospital on April 26, 2009. For the first cases the laboratory tests were PCR amplification of the matrix (M) gene followed by sequence analysis which demonstrated a perfect match to the A/California/7/2009 strain [9]. Later cases were identified using the WHO/CDC protocol (http://www.who.int/csr/resources/publications/swineflu/CDCRealtimeRTPCR_SwineH1Assay-2009_20090430.pdf). The clinical samples collected were throat swab specimen and two nasal swab specimens, one from each nostril collected into viral transport medium.

A database of all cases confirmed to have swine flu was assembled at the National Influenza Center in the Central Virology Laboratory, Tel Hashomer, Israel, from the 26^th ^of April until July 7, 2009. All patients tested were requested to provide the following personal details: name, age, sex, disease initiation (as reported by the patient), date of arrival to the clinic, return from a foreign country in the previous week, contact with other infected patients, visits to educational institutions, and symptoms. Patients were also classified according to the respective social groups to which they belong to such as soldiers, ultra-orthodox Jews, or Arabs. Altogether circa 2,400 samples of patients with symptoms of influenza like illness (ILI) were taken, of which 713 (30%) were found positive. This number should closely match most Israelis with symptomatic swine flu in this time period (see below for more details). As a result of the WHO guidelines, the systematic collection of samples from all suspected patients ended after July 2 2009, and the analyses reported here are based on data collected up to July 7.

Obtaining a comprehensive database that includes most symptomatic cases was possible due to both extensive efforts made by the Israeli health authorities and particular attributes of the country of Israel. During the initial phase of the outbreak, the Deputy Director General of the Ministry of Health (MoH) conducted meetings or teleconferences with representatives of all medical institutions, government agencies responsible for disease control and professional consultants regarding the pandemic situation almost daily. The goal of the MoH during this period was to mitigate the epidemic as much as possible. An important part of the MoH strategy was based on having the best possible surveillance of infected cases. To achieve this, the General Director of the MoH published between the 5^th ^of April and the 17^th ^of August 7 MoH guidelines and binding regulations for the H1N1 pandemic. The documents have a legal status and the entire Israeli medical establishment (doctors, Health Maintenance Organizations - HMOs, hospitals) is required to follow these guidelines. In addition the Israeli authorities launched a media campaign which called for every individual with influenza like symptoms, or anyone in contact with people having ILI symptoms, to be tested for the H1N1 virus. Below we summarize their main policy guidelines.

On the 5^th ^of April 2009, the Ministry added H1N1 to the list of mandatory reportable diseases. Anyone suspected of having a respiratory illness, was tested using swabs which were sent to the Ministry's Central Virology Laboratory. In the first stages of the outbreak (until 17.6.2009) all laboratory diagnosed cases were hospitalized. Official epidemiological forms were provided to the medical staff at hospitals and in community clinics. In cases of a cluster of three or more confirmed cases the guidelines required the Health District Physician to conduct a full epidemiological investigation and to report the results to the MoH headquarters. At the Ben-Gurion international airport (the main international airport in the country) a special medical clinic was opened for examining every passenger who arrived to Israel from Mexico within 7 days of departure from Mexico. At a later stage every passenger arriving from Mexico, the US or Canada with fever within the previous 24 hours was tested and sent to a hospital directly from the airport. All patients were hospitalized in isolation conditions to prevent further spread of the disease.

In addition to the above, Israel has several unique characteristics which made a major contribution to the success of MoH's surveillance during the initial stage of the pandemic. Israel is a small country in size (21,000 km^2^). There is hardly any human movement across the land borders and most people entering the country are passengers entering the Ben-Gurion International airport. Also, during the initial phase, the pandemic received a great deal of attention from all branches of the media. The Israeli public health system is composed of four HMOs which work in tight connection with the MoH and there was excellent collaboration during the outbreak between the different organizations. Israel also has wide experience in preparing for catastrophic events as evidenced by the detailed Israeli pandemic preparedness program from 2007 (http://www.health.gov.il/Download/pages/tol_pand07.doc) which had planned the surveillance program in great detail ahead of time. Taking into account all of these attributes, the Israeli MoH argued that during the initial phase of the epidemic, it was possible to pinpoint most symptomatic case of swine flu is Israel - making this dataset very promising for epidemiological analysis.

Estimating the reproductive number *R*_*0 *_is of great importance when studying epidemics, When, as in many situations, the population is not fully susceptible (*S*_*0 *_< N) the effective reproduction number - *R*_*e *_which is defined as: *R*_*e *_*= R*_*0*_·*S*_0_/N [10], is used. Our estimates of *R*_*e *_are based on the formulation of a discrete-time stochastic epidemic model closely related to the well known SIR model [11]. The model allows for intrinsic demographic stochasticity and makes a clear distinction between primary and secondary infectors (see also [12]). Full details describing the derivation of the estimate for *R*_*e *_can be found in [13]. For the purposes of this paper we need only state our formula, namely:(1)

Here *i(t) *represents the number of newly infected individuals on day *t *as observed in the surveillance time-series which are to be differentiated from primary or imported infectors arriving from abroad on day *t *as denoted by *i*^*0*^*(t)*. The numbers *P*_*τ *_*(1*≤*=τ*≤*=d) *represent the generation time distribution or infectivity profile. In a totally susceptible population, *P*_*τ *_is the fraction of infections generated by an infective person which occur on day *τ *of infection (thus). The generation time distribution is estimated from the infection networks data as described below.

The data provides the number *i(t)*of confirmed cases on each day *t *of the study period. Since the cases are all laboratory-confirmed, there is little danger of false-positives. However, the number *i(t) *may under-estimate the true incidence, for two reasons: some of the infections may be non-symptomatic, and some of the symptomatic infections may not seek medical care. However, under the assumption that the probabilities of non-symptomatic infection and of seeking medical care are constant during the period in question, if there is an under-estimation of the true number of cases, it will not affect the estimate of the effective reproductive number. Indeed, denoting the probability that an influenza case will be symptomatic by *s *and the probability that a symptomatic case will be reported by *r*, then the true number of cases  is given in terms of the confirmed number of cases by . Since the estimator of *R*_*e *_does not change when the time series *i(t) *is multiplied by a constant, the estimate is unchanged.

A major advantage of estimating *R*_*e *_by fitting a stochastic model to the data is the fact that we do not make any a-priori assumption of exponential growth. Although the stochastic model indeed leads to exponential growth when the number of cases is large, the model also generates an initial "stochastic" phase, and is thus suitable for modeling the initial phase of an epidemic.

There were 451 patients who reported the date at which they perceived their illness began. We calculated the difference between this onset date and the date in which they arrived at the health clinic or hospital for treatment. Also, most individuals tested for swine flu were asked by their doctor to identify who they believed they were infected from. Of the 713 cases, 183 (25.6%) were able to provide information establishing contact links. This data was used to assemble contact networks which map the connections between an infected person (infector) and the different individuals he/she infected (infectees). Based on the contact networks, it was possible to determine the generation-time interval distribution for swine flu - the distribution of durations between the time an individual becomes infected and the times of infection of the people he or she infects. We used the reported dates of disease initiation at both ends of a link as the samples of the generation-time intervals. We counted all infector → infectee relations between males (M) and females (F), and also between age classes. The expected numbers of these interactions were then calculated assuming random mixing between groups of infectors and infectees via standard contingency table analysis [14].

We used the geographic locality information to plot the spatial dynamics of the epidemic. Each case was assigned to one of fifteen sub-districts in Israel. As the correlation between the population size and the number of cases in each sub-district proved highly significant (P-value <0.0001, R^2 ^= 0.833), for all further analyses we used the rates of swine flu per 100,000 people. These rates were plotted for each week of the epidemic in the different sub-districts. We conducted a spatial autocorrelation analysis by calculating the global Moran's I index [15] for the above rates in the first 11 weeks of the epidemic. The index was also calculated for the cumulative rate over the entire time frame.

We conducted a forward stepwise multiple regression of the influenza rates (in the different sub-regions), against several explanatory variables: sub-district area, percentage of built area, average gross household income, average number of inhabitants per household, percentage of workforce arriving from other sub-districts, and mean maximum and minimum temperatures over the duration of the epidemic. Furthermore, we applied the Akaike information criterion (AIC) in order to choose the best fitting model among several possible models (see below) [16].

## Results

**Figure 1:**
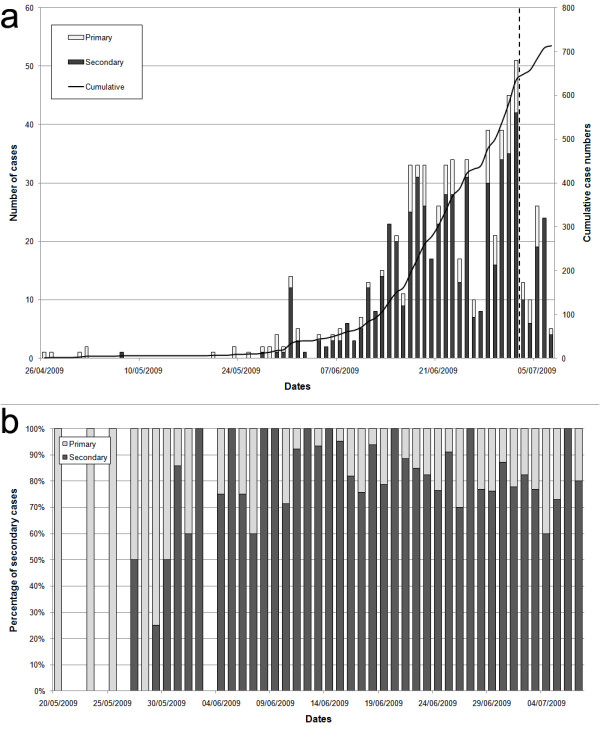
**Time series of swine flu in Israel**. a- time series of swine flu cases in Israel between the 26/4/2009 and the 7/7/2009, altogether 713 cases. Bars represent the incidences per day (left Y-bar scale) and line the cumulative number (right Y-bar scale). Also marked is the date when the systematic data collection ended (dashed line). b- Percentage of primary versus secondary cases over the epidemic from the 20^th ^of May onwards.

**Figure 2:**
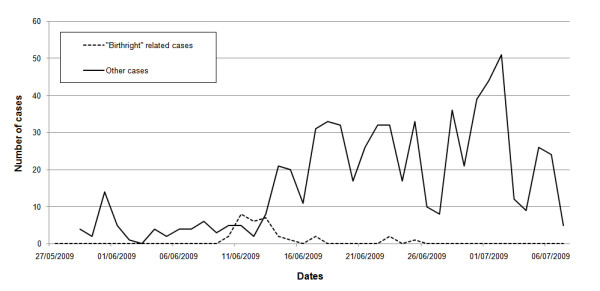
**"Birthright" influence on spread**. Influence of the group of young American students (from the "Birthright" project) on time series of swine flu cases in Israel. The dashed line shows all the cases that were either group members or identified themselves as being in contact with the "Birthright" group. The solid line displays the rest of the cases.

The time series of swine flu cases in Israel during the initial phase of the outbreak is displayed in Figure 1a. In the first weeks of the epidemic, nearly all new cases arrived sporadically from abroad, infecting some individuals but eventually becoming locally extinct, as seen in the period April 26 to June 6, 2009 (Figure 1a). In the month of June the percentage of primary cases (arriving from abroad) vs. secondary cases (infected locally) drops, but still remains relatively high (Figure 1b). In the second week of June, some nineteen members of a group of visiting young American students (from the "birthright" program) traveling the country were confirmed to have swine flu. The records show that at least twelve other patients came into contact with this group, most of them soldiers (see below). Since the arrival of these young travelers, there was a clear jump in the number of cases and in the spatial spread of the disease (Figures 1a, 2). Although the pandemic was inevitable in Israel, with or without this specific group, the surveillance procedures were refined to a degree that enabled identification of the key triggering factors.

### Basic reproductive number

For the Israeli swine flu time series (Figure 1), our modeling approach produced estimates for *R*_*e *_= 1.06 with a 95% confidence interval 0.963-1.134. Our results highlight the importance of separately modeling imported infected persons migrating into a region or country, rather than considering them as just a particular subset of the local infected population. The latter is an incorrect approach and will result in an over-estimate of *R*_*e*_. For example, had all infections been regarded as local, the estimate for *R*_*e *_via Equation (1) (taking *i*^*0*^*(t) *= 0), would have resulted in *R*_*e *_= 1.27 (95% C.I. 1.17,1.37). Alternatively, if the imported infectives had been removed from the data, this would have resulted in *R*_*e *_= 1.26 (95% C.I. 1.16, 1.37). Although as the epidemic spreads the number of immigrant cases becomes negligible in comparison to the locally infected cases, in the timeframe covered by this study this stage is not yet reached (see figure 1b). As the example here shows, at the initiation of the epidemic, the imported infectives have a significant effect on the estimate of *R*_*e*_.

**Figure 3:**
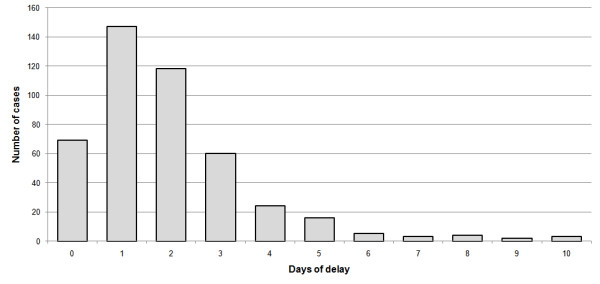
**Delay between disease initiation and doctor visit**. Frequency distribution of the number of days of delay between the reported initiation of the disease and the visit to the health clinic or hospital (N = 451).

The mean time of delay between arrival at the hospital or health clinic and the reported date at which patients perceived their illness began is 1.93 days with a mode of one day and median of two days (see Figure 3). Hence, a large portion of the patient's infectivity period was in fact prior to their diagnosis. The significance of this finding is discussed below.

### Age distribution

**Figure 4:**
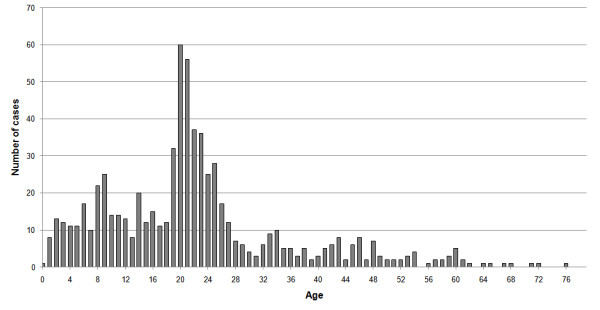
**The number of swine flu cases at each age**.

**Figure 5:**
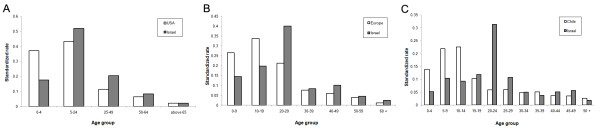
**Age distribution of cases in different countries**. Standardized rates of swine flu in different age classes and different countries. The number of cases in each age class is divided by total population in these ages, and these rates are later divided by the sum of all of the rates in all age classes. These rates were calculated for the USA - A (confirmed cases from the 15/4-24/7/2009 Based on [55]), Europe - B (confirmed cases up to the 20/7/2009 based on [17]) and Chile - C (all confirmed cases up to the 2/7/2009 based on [7]) and compared to their parallel rates in Israel.

**Figure 6:**
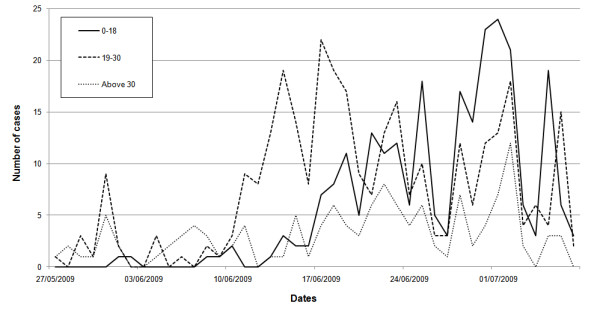
**Time series of swine flu cases divided into three different age groups**.

The mean age of the confirmed cases was 22.1 years with a median of 21 and a mode of 20. The age distribution of cases in many other countries in the same period gave a median age lower than 20 [17] (Figure 4 shows the age distribution of cases in Israel). Cases were divided into three different age classes 0-18, 19-30 and above 30. Calculating the incidence rate for the secondary cases (i.e. those infected within Israel) over the entire period of data collection for each of the age groups, we obtained the following rates per 100,000: 7.5 (0-18), 17.3 (19-30), 2.9 (31+). Two features of this age distribution seem striking: 1) The very low rates in adults over 30 compared to the other groups. 2) The high rates among young adults (19-30) compared to children (more than twice). Compared to data from other countries (see Figure 5) it appears that the first feature is common to many countries, while the second feature is not typical. The 19-30 age group rapidly increased from early June and remained high, but the 0-18 age group became significant only later (Figure 6).

### Contact Structure and Infection Networks

Altogether there were 66 separate infection networks totaling 183 nodes, with 123 links between them, many networks were disconnected with many isolated links. 55.2% of the nodes had no outgoing links, 33.3% had one outgoing link, 7.1% had two outgoing links and 2.7% had three outgoing links. In addition there were three patients who might be considered "superinfectors" with four, seven and ten outgoing links. The mean number of outgoing links per node was 0.67. Note that this number is much lower than the value of *R*_*e *_= 1.06 we calculated for this timeframe, which is unsurprising in view of the fact that many of our networks only provide a sample of all infections. Despite the fact that our networks only represent a sample of actual infections we can, under the assumption of random sampling, use them to estimate statistical characteristics of infection times and relative rates of infection among various groups.

### Generation-time distribution

**Figure 7:**
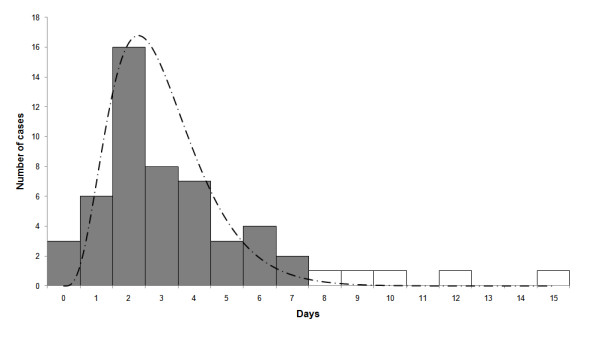
**Frequency distribution of the swine flu generation time**. This is based on differences in reported disease initiation dates that were calculated from our infection networks. Also displayed is a Gamma distribution curve (dashed line) based on data only up to seven days - dark bars (see text for details).

The mean generation-time was found to be μ = 2.92 and its standard deviation σ = 1.79 based on a generation time distribution of up to seven days. Figure 7 displays the frequency histogram of the generation-time. Superimposed on the histogram is the best fitting gamma distribution which was found to have μ = 2.95 and σ = 1.43 (with the parameters a = 4.25, b = 1.44). The contact networks include reports of infection for periods longer than seven days. However, data about infections beyond seven days is controversial [18], and were not included when fitting the gamma distribution.

### Male and Female Contact rates

The networks were composed of 123 different links defining infector → infectee relations between males (M) and females (F) (Table 1). Our analysis shows that the observed numbers are not significantly different than expected by random mixing (p = 0.942, χ^2 ^= 0.0052, Yates corrected chi-square test).

**Table 1:** Characterization of all possible observed male-female interactions

		Infectee		
		male	female	**Sum**.
Infector	**male**	41 (40.32)	46 (46.6)	87
	**female**	16 (16.68)	20 (19.31)	36
	**Sum**.	57	66	122

For example there were 57 infected males, of which 41 contracted influenza from male infectors, while 16 contracted influenza from female infectors. The numbers given in brackets are the expected number of interactions determined from a standard contingency table analysis.

### Age-class contact structure

The contact networks analysis of the age-class structure is shown in Table 2. The observed data is significantly different from expected (p = 6.8E-6, χ^2 ^= 29.3, chi-square test) and individuals in different age classes are not mixing randomly. The most significant differences are found within age-classes (i.e., along the matrix's diagonal) which show large deviations from random mixing. Individuals within the 19-30 age class infect one another three times more than expected via random mixing. The two age-groups 0-18 and 19-30 appear to infect one another less than expected (see Table 2).

**Table 2:** Comparison of inter and intra age-group infections (3 age group classes)

		Infectee			
		0-18	19-30	above 30	**Sum**.
	**0-18**	46 (36.1)	6 (13.7)	18 (20.1)	70
infector	**19-30**	6 (14.9)	15 (5.7)	8 (8.3)	29
	**above 30**	11 (11.9)	3 (4.5)	9 (6.6)	23
	**Sum**.	63	24	35	122

We use our infection network to compare observed vs. calculated rate (using all our data for null probabilities). The matrix displays the number of observed versus expected (in brackets) values between all combinations of age groups.

### Sex ratio

The male to female sex ratio of swine flu cases was skewed, with 59.6% males and 40.4% females (n = 713). This is significantly different from the sex ratio of the entire Israeli population which stands at 50.4% males to 49.6% females (P < 0.0001, χ^2 ^= 24.1, Yates corrected chi-square test after appropriately normalizing the general population by the age structure distribution of swine flu cases). The high male sex ratio was compared to that observed in seasonal flu records collected over a ten year period from one of Israel's health maintenance organizations (n = 286,310). The seasonal flu data has more females (46.1% males, 53.9% females), and these proportions are significantly different from those of swine flu (P < 0.0001, χ^2 ^= 52.18, Yates corrected chi-square test). The Israeli male to female sex ratio for swine flu cases is also higher than that reported in Europe (male 52.4%, female 47.6% - [19]) and several other countries with available data (Belgium -[20], Chile -[21], France - [22], Italy -[23], Greece -[24]). The analysis of a more updated swine flu data-set which includes an additional 3,340 confirmed cases further supported this deviation with a male percentage of 54.5%.

### Social groups

We examined the epidemic dynamics of several distinguishable social groups. Soldiers, for example, who comprise some 2.4% of the Israeli population [25], are relatively connected within themselves and isolated from the rest of the population in terms of daily activities. Soldiers were highly represented among swine flu cases with 107 confirmed cases, which is an incidence of 60.6 cases per 100,000 people in this group; seven times higher than the non-soldier public (8.5 cases per 100,000 people). Ultra-orthodox Jews were found to have roughly the same incidence as the rest of the Israeli population (10.4 versus 9.8 cases per 100,000 respectively). Nevertheless, the first ultra-orthodox swine flu case was reported on June 22, 2009, and since this date ultra-orthodox Jewish cases represent 17.8% of all cases (in this, more limited time frame), whereas they represent only about 10% of the Israeli population [26]. In our database there were only 3 cases of Arabs with swine flu. This value corresponds to an incidence of 0.2 cases per 100,000 people, compared to 12 cases per 100,000 people in the non-Arab population of Israel.

### Spatial analysis

**Figure 8:**
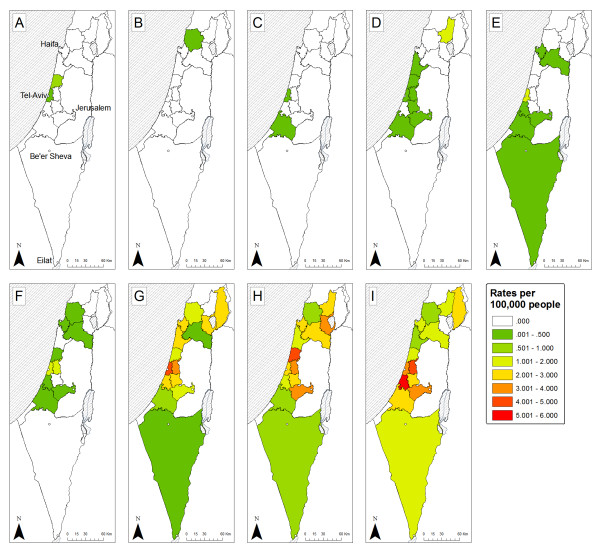
**Spatial spread of swine flu in sub-districts of Israel**. The data are combined for each week up to week ten of the epidemic (week three had no cases in it and therefore isn't displayed). The color of the region represents its rate of Influenza, from dark green - low rate (up to 0.5 cases per 100,000 people) to red- high rate (5-6 cases per 100,000 people). The panes represent different weeks (A-first 26/4-2/5/2009, B-second 3-9/5/2009, C-fourth 17-23/5/2009, D-fifth 24-30/5/2009, E-sixth 31/5-6/6/2009, F-seventh 7-13/6/2009, G-eighth 14-20/6/2009, H-ninth 21-27/6/2009, I-tenth 28/6-4/7/2009). Also on the map five major cities of Israel are marked as dots; the cities are (from north to south): Haifa, Tel-Aviv, Jerusalem, Be'er-Sheva, Eilat.

The rates for each week of the epidemic in the different sub-districts are displayed in Figure 8. The epidemic is more pronounced in the central-western part of Israel, which generally showed higher rates than other regions and from there it spread to the north east, and south. In the first six weeks, swine influenza was unable to persist in all regions; it went locally extinct in 7 of the 13 sub-districts up to this point. Additionally the incidence rates remain below 1 infected person per 100,000 people. After this period the epidemic climbs - regions which had infections remained infected, and the rates increase.

Table 3 displays the Moran's I values for the autocorrelation analysis. In the first six weeks there is a pattern of spatial non-random dispersion - negative (but non-significant) I values. From the seventh week onward the pattern changes, the rates in the different regions show autocorrelation - positive I values which are significant for 3 of the 5 weeks (see Table 3). The total Moran's I value for all of the weeks combined also showed significant spatial autocorrelation (I = 0.194, Z = 4.267, P < 0.0001).

**Table 3:** Global spatial autocorrelation computations using Moran's I values

Week	Moran's I	Z value	P value
1	-0.035	0.398	0.690
2	-0.058	0.107	0.915
3	-	-	-
4	-0.023	0.616	0.538
5	-0.143	-1.019	0.308
6	-0.103	-0.563	0.573
7	0.176	2.752	0.006
8	0.141	2.061	0.039
9	0.004	0.675	0.500
10	0.401	4.787	<0.001
11	0.066	1.299	0.194
All weeks	0.194	4.267	<0.001

Displayed are the values of Moran's I values calculated based on rates and distances between sub-districts of Israel, for each of the initial 11 weeks of the epidemic (but the third week that had no cases). Also displayed is this value for the cumulative rates of all of the weeks. For each value of Moran's I we show the corresponding Z and P values.

When analyzing the various explanatory factors of the swine-flurates in different sub-districts, using the stepwise approach, only two factors stood out as significant - percentage of built area and gross household income (P-value = 0.0001, R^2 ^= 0.77). These two spatial predictors are significantly positively correlated to each other (P-Value = 0.046, R^2 ^= 0.273). When looking at the AICc values of 17 different model combinations, the model including just these two factors had the lowest AICc score with an AICc weight of 55.3% (see Table 4 for a detailed table of AICc and model combinations).

**Table 4:** Values of AICc for models with different variables explaining spatial distribution of influenza in sub-districts of Israel

Model parameters	K	AICc	Delta AICc	AICc weights	Cumulative weights	P-value	**Adjusted R**^**2**^
Built area + income	4	1.16	0	0.55	0.55	0.000145	0.7327
Built area	3	3.81	2.66	0.15	0.7	0.000299	0.6201
Built area + income + household size	5	5.48	4.32	0.06	0.76	0.000676	0.7151
Built area + income + commuting	5	5.82	4.67	0.05	0.82	0.000766	0.7084
Built area + commuting	4	6.33	5.17	0.04	0.86	0.001147	0.6226
Commuting	3	6.75	5.59	0.03	0.89	0.001123	0.5378
Income + commuting	4	7.19	6.03	0.03	0.92	0.001614	0.6004
Built area + household size	4	7.59	6.43	0.02	0.94	0.001897	0.5895
Household size + commuting	4	8.11	6.95	0.02	0.96	0.002332	0.5752
Income	3	8.48	7.32	0.01	0.97	0.00247	0.4814
Income + household size	4	9.02	7.86	0.01	0.98	0.003359	0.5485
Income + household size + commuting	5	9.98	8.83	0.01	0.99	0.003381	0.6152
Built area + household size + commuting	5	10.97	9.81	0	0.99	0.004787	0.5892
Built area + income + household size + commuting	6	11.25	10.09	0	1	0.002689	0.6879
Household size	3	12.45	11.29	0	1	0.01569	0.3242
Temp	3	15.77	14.61	0	1	0.0798	0.1572
Built area + income + household size + commuting + temp	7	18.24	17.08	0	1	0.007742	0.6648

The analysis was conducted on 15 subdistricts with combinations of five explanatory variables (see text). The table displays the combination of the models chosen, the number of parameter in each model (K), and their appropriate AICc, Delta AICc, AICc weights, cumulative weights, P-values and adjusted R^2 ^values.

## Discussion

Pandemic influenza is considered one of the largest burdens to human health [27]. However, data on the initiation phase of pandemic is scarce. Understanding the dynamics at this phase is fundamental in determining the consequent impact of the disease on human populations. The data gathered in Israel during the initiation of the current H1N1 influenza pandemic is one of the best such datasets in the world, creating a rare opportunity for analysis. Such data and real time analysis can greatly aid management schemes.

It is now known that both for seasonal influenza [28] and the swine flu pandemic there was a considerable proportion of asymptomatic cases [29-31]. However it is unclear how important, if at all, is the role of these cases in epidemic dynamics [32,33], The proportion of asymptomatic cases in the 2009 pandemic is believed to range from 10% to 90% [29,30]. Unfortunately in the current study we were unable to analyze the role of the asymptomatic cases on the spread of the pandemic. However, if the proportion of symptomatic to asymptomatic cases is constant during the period of the study then our estimates of *R*_*e *_should be valid (see method for details). Furthermore, our epidemiological analyses of rates in different social groups, ages, sexes or regions should also hold providing asymptomatic cases are distributed in the population in the same manner as symptomatic cases. Therefore this lacuna in our data should not undermine the conclusions based on it.

Our estimate of the reproductive number *R*_*e *_= 1.06 was only slightly larger than unity. This is relatively low compared to calculations reported in Mexico, USA, Canada, Australia, New-Zealand and Japan based on data from the initiation phase of the current epidemic which range from 1.31-4.11 [34-40], and also lower than estimates based on data from previous pandemics [41]. However, low estimates of *R*_*e *_were also reported for Mexico and the Netherlands [42,43]. This low estimate could derive from either unfavorable weather condition upon the late arrival of this strain [44], more immunity of the Israeli population to this particular strain, relative to other countries [45,46], or efficient containment measures by the Israeli health authorities. The fact that *R*_*e *_was so close to unity raises possibility of containing or mitigating the spread of the disease [47].

Many studies of the current pandemic emphasize the lower rates among adults older than 30 years relative to the younger age groups, and the same was found here. However, an intriguing feature of the age distribution derived from the Israeli data is the very high rates among young adults (19-30) as compared to children. This characteristic appears to be exclusive for Israel. We should mention that during the time of the research there were no prolonged school vacations and the school year ended either on the 30^th ^of June for primary schools or the 20^th ^of June for high-schools (i.e., at similar dates to other northern hemisphere countries). With respect to school closure - only one school in Israel was closed, for a few days, as a response to a high ratio of infection in one of its classes and not as a preplanned preventative measure. We can therefore assume that the different age morbidity in Israel has no clear relation to any events that pertain to the Israeli education system. Ahmed et al. [6] show that during the 1918 pandemic there were higher infection rates in young adults, which were explained by more immunological memory for adults (aged > 30 ) and a "honeymoon period" for children having less severe symptoms. It is possible that a unique memory effect has a role in the current pandemic in Israel, shaping its age group distribution.

It is also possible that this effect is due to transient dynamics at the start of the epidemic related perhaps to the higher representation of young adults, as compared to children, among international travelers. Thus the age distribution at the initial stages of an epidemic can be quite different from the distribution at later stages due to the importance of stochastic effects combined with population heterogeneity. Therefore age-class data from the initial phase of outbreak should be used with great caution in making decisions, for example on vaccination policy.

The contact network data enabled us to estimate the generation-time interval distribution, as well as the matrices describing infections among sexes, and age classes. An analysis of the contact data shows that the infectivity of most people peaks within two days followed by a gradual decline with instances of influenza transmission even a week after the infector's disease initiation. This information should be coupled with the fact that it also takes several days (with a mean of 1.9 days) for infected people to reach a clinic for diagnosis in the first place (other countries show similar patterns [22-24]). This fact can make control of the spread of the epidemic difficult as most of the infection occurs prior to diagnosis.

High levels of heterogeneity in transmission were observed when comparing different groups such as ultra-orthodox Jews, soldiers, Arabs, and by looking at sexes. For example, after the initial infection of the ultra-orthodox community (June 22^nd^) their infection rate was higher than the general population. This community is relatively isolated from the rest of the population, which may account for the delay in its infection. However, ultra-orthodox Jews live in very dense communities with relatively big households [26] which would lead to higher rates once infection commenced. A similar mechanism can also be postulated for the soldier sub-group - which has many internal connections. A group of soldiers living in close quarters in the USA have also exhibited increased swine flu illness exposure and susceptibility [48]. In Israel, soldiers were infected early on in the epidemic via contacts with a group of American students and it is understood that this chance initial event combined with their unique living quarters and behavior, is likely to be the explanation for their high infection rates. All of these are possible explanations for the unusual age distribution found among the infected Israeli population, as the soldiers all belong to the 19-30 age group.

In contrast, the Arab population - another relatively isolated social group within Israel - showed significantly lower rates of infection throughout. It is possible that during the timeframe of this study the disease had yet to penetrate this social group. These results can demonstrate how in the initial phase of an outbreak - within distinct social groups - different social groups can present different behaviors due to chance infection events. Another puzzling result concerns the male bias in the sex ratio of infections, a result also noted for other infectious diseases [49]. It is hypothesized that behavioral and physiological differences could result in this gender bias [50,51]. Making use of this knowledge of how heterogeneity affects disease dynamics might be useful in improving control, mitigation and policy programs [52].

The spatial analysis reveals that percentage of built area and gross household income explain differences in the prevalence of swine flu between regions. The first of these factors pinpoints the epidemic to those regions which are more densely populated - an attribute that can aid the rapid transmission of influenza. Higher income families are inclined to travel more and come in contact with more people, both internally and abroad [50,53]. Thus, it is likely that persons from such families are more likely to come in contact with an infected individual in the initial stages of an international outbreak.

The central coastal plain of Israel, in and around the city of Tel-Aviv, has consistently higher rates and perhaps acts as an initiation point for the spread to other regions. This is also manifested by looking at the values of the spatial autocorrelation (Table 3). The central coastal plain of Israel is the cultural and business hub of the country; it is the most densely populated region, with more commuting into it from neighboring regions [54]. This region also houses the major international airport of Israel, harbors most of the commerce and trade of the country, and had milder temperatures during these eleven weeks. It is therefore unsurprising that there are higher rates of swine flu in this region as well as it being an initiation point of the spread of the epidemic to other regions in the country.

## Conclusions

Our detailed contact networks enabled us to perform unique analysis on the particular relationships between different groups of society (categorized by age, sex, ethnicity, etc.). The analysis highlighted at-risk groups as well as paths of spread between them, at this initial stage. As contact data is generally not available, few such studies have been conducted. Such detailed information, if obtained and analyzed in real time has much potential in management and mitigation of an epidemic as it spreads.

Our analysis made it possible to elucidate the spatio-temporal dynamics of the initiation of this pandemic in Israel. Examining the rates of influenza in different regions of Israel as the pandemic developed uncovered patterns of spatial spread, as well as disease clusters. Correlating disease rates to various socio-economical attributes of these regions suggested a potential mechanism generating these spatial patterns. Such spatial analysis holds much promise for other epidemiological studies with available locality information.

Gathering detailed data from different parts of the world and looking for statistical universalities as well as crucial differences should be of great value to better characterize and improve our understanding of the initial phase of the swine flu pandemic and forthcoming outbreaks. Further work is also needed to evaluate the next phase of the pandemic. We hope our contribution both sheds light on the dynamics of swine flu and provides methods for analyzing future pandemics.

All authors declare no conflict of interests relating to this article.

## Authors' contributions

MM, EM collected the data, and performed the laboratory tests, OB, UR, AH, RY, GK, LS constructed the database, UR, LS, AH, RY, GK & EM conceived and designed the experiments and analysis, UR, RY, OB, AH & GK analyzed the data, UR, AH & LS wrote the paper

## Pre-publication history

The pre-publication history for this paper can be accessed here:

http://www.biomedcentral.com/1471-2334/11/92/prepub
